# Sexual communal motivation in couples coping with low sexual interest/arousal: Associations with sexual well-being and sexual goals

**DOI:** 10.1371/journal.pone.0219768

**Published:** 2019-07-17

**Authors:** Jacqueline V. Hogue, Natalie O. Rosen, Amanda Bockaj, Emily A. Impett, Amy Muise

**Affiliations:** 1 Department of Psychology, Faculty of Health, York University, Toronto, Ontario, Canada; 2 Department of Psychology & Neuroscience, Faculty of Science, Dalhousie University, Halifax, Nova Scotia, Canada; 3 Department of Psychology, University of Toronto Mississauga, Toronto, Ontario, Canada; Ruhr-Universität Bochum, GERMANY

## Abstract

Women coping with female sexual interest/arousal disorder (FSIAD) report lower sexual and relationship satisfaction compared to healthy controls. In community samples, high *sexual communal strength* (i.e., the motivation to meet a partner’s sexual needs) is associated with higher sexual desire and satisfaction, but high *unmitigated sexual communion* (i.e., the prioritization of a partner’s needs to the exclusion of one’s own needs) is associated with lower sexual satisfaction. People higher in sexual communal strength report engaging in sex for *approach goals* (i.e., to enhance intimacy in their relationship), but not for *avoidance goals* (i.e., to avert conflict or a partner’s disappointment) and this is one reason why they report greater sexual desire. In the current sample of 97 women diagnosed with FSIAD and their partners we investigated the association between sexual communal strength and unmitigated sexual communion and sexual well-being (i.e., sexual desire, sexual satisfaction and sexual distress) and sexual goals (i.e., approach and avoidance goals). Women who reported higher sexual communal strength were more likely to pursue sex for approach goals and their partner reported greater sexual satisfaction. When partners reported higher sexual communal strength, they reported higher sexual desire, but when they reported higher unmitigated sexual communion, they reported higher sexual distress. Additional associations emerged for couples who engage in sex more (compared to less) frequently. Our findings demonstrate that being motivated to meet a partner’s sexual needs is associated with greater sexual well-being for couples coping with FSIAD, but when this motivation involves neglecting one’s own needs, people do not report greater sexual well-being and instead, partners report higher sexual distress.

## Introduction

Low sexual desire is a common complaint, particularly among women [1]. In large scale, nationally representative surveys, nearly a quarter of women report low sexual desire lasting several months over the past year, and for 7% to 30% of women, low sexual desire is accompanied by significant distress [2–4]. Female Sexual Interest/Arousal Disorder (FSIAD) is the clinical diagnosis for a female sexual dysfunction characterized by low sexual desire and/or arousal accompanied by distress, and which is not better accounted for by another medical or psychiatric condition [5]. For a diagnosis of FSIAD, women must report reduced or low levels of at least three of the following symptoms during at least 75% of their sexual encounters and for at least six months: desire for sex, sexual fantasies/thoughts, initiation and receptivity of sexual activity, sexual pleasure, desire elicited by sexual stimuli, and/or genital or non-genital sensations [5]. Etiological models of FSIAD acknowledge the importance of interpersonal factors [6] in the maintenance of low desire and associated distress, and couples therapy is frequently a first-line intervention [7]. However, we know very little about the interpersonal factors that might be protective for women’s low desire and FSIAD couples’ associated difficulties.

Women coping with FSIAD report lower health-related quality of life, including more depressive symptoms, and lower sexual and relationship satisfaction compared to healthy controls [8–10]. In fact, women with FSIAD who are partnered are five times more likely to report sexual and relationship distress compared to affected women who are unpartnered [4], underscoring the interpersonal context of the disorder. Although few studies have included the partners of women with FSIAD, the existing research suggests that partners report negative consequences as well. Compared to partners of women not coping with a sexual dysfunction, partners of women with FSIAD report lower sexual and relationship satisfaction and more sexual distress [10]. In an early small study of 40 couples, couples coping with low desire (*N* = 20) reported a more limited sexual repertoire and less pleasure and enjoyment during sex than healthy controls (*N* = 20) [11], as well as more frequent sexual disagreements and dissatisfaction with their frequency of sexual activities [12]. In a qualitative study, both partners in couples coping with low desire tended to blame each other for the problem [13].

While no single cause of FSIAD has been identified [14], risk factors for the development and maintenance of FSIAD include biological, psychological, interpersonal, and sociocultural factors [6]. Therefore, a biopsychosocial approach to assessment and treatment that takes relationship factors as well as the woman’s partner into account has been recommended [6,14]. Despite these recommendations and the fact that low sexual desire and arousal frequently occur in the context of a romantic relationship, there has been limited focus on the role of interpersonal factors [4]. However, there is promising, initial evidence that interpersonal factors, such as relational and partner-focused sexual motivational factors, play an important role in maintaining sexual issues in couples coping with a sexual dysfunction [4,12,15]. For example, in a community sample of women—about half of whom reported sexual dysfunction—perceptions of poor relationship quality or of a partner’s sexual dysfunction were connected to women’s low desire/arousal [15]. Given that both partners report negative consequences of FSIAD [10] and partnered women with FSIAD report greater distress than their unpartnered counterparts [4], additional research investigating the relational and motivational factors that maintain these sexual issues and the well-being of both partners is crucial for the further development of interventions for couples coping with FSIAD. In the current research, we investigate the role of two novel interpersonal factors—*sexual communal strength* (SCS; i.e., the motivation to meet a partner’s sexual needs) and *unmitigated sexual communion* (USC; i.e., a focus on meeting a partner’s needs to the exclusion of one’s own needs)—in the sexual well-being (i.e., sexual desire, sexual satisfaction, and sexual distress) and sexual goals (i.e., approach and avoidance sexual goals) of affected couples [16].

### Sexual communal motivation and sexual well-being

Women with FSIAD in relationships often continue to engage in sex despite experiencing low desire [17], and their motivations for doing so may be associated with both partners’ sexual well-being. Theories of sexual communal motivation suggest that responsiveness to a partner’s sexual needs (i.e., high sexual communal strength; SCS) even during times when partners have different sexual desires can have benefits for romantic relationships [18,19]. Associations between SCS and sexual and relationship well-being have been examined in experimental [18], longitudinal [19], and daily experiences studies [19]. In a sample of couples in long-term relationships, people higher in SCS reported higher daily sexual desire and were more likely to maintain higher sexual desire over time [16]. Even among couples coping with a sexual dysfunction (e.g., genito-pelvic pain/penetration disorder), higher SCS is associated with better sexual function, of which one component is sexual desire, for both partners [20]. More intuitively, people with partners higher in SCS feel more satisfied in their relationships and more committed to maintaining their relationship over time, compared to people with partners lower in SCS [19]. In fact, in both community [21] and clinical samples of women coping with a sexual dysfunction [20], on days when one partner reported higher SCS, the other partner reported greater sexual and relationship satisfaction. One reason that women with low desire report for engaging in sex is to make their partners happy [17] and the extent to which this is communally motivated may be associated with sexual well-being.

Previous findings also suggest that SCS can help couples navigate sexual discrepancies or maintain sexual and relationship satisfaction during times when sexual desire is low. People in romantic relationships who were higher in SCS were motivated to meet a partner’s sexual needs even on days when they experienced lower sexual desire than their partner and in turn, both partners reported higher sexual and relationship satisfaction [18]. In a sample of new-parent couples—a time when desire and satisfaction tend to decline and sexual problems are more likely to arise [22,23]—having a partner who was higher in SCS was associated with greater sexual and relationship satisfaction for both new mothers and new fathers [24]. However, we do not yet know whether SCS will be associated with sexual well-being in couples coping with chronic, distressing sexual desire, as is the case with couples coping with FSIAD. In a qualitative study, one strategy that women report engaging in to manage desire discrepancies in their relationship includes trying to understand or meet their partner’s needs, which closely parallels SCS, and they report this strategy to be at least somewhat helpful in better navigating differences in desire [25]. Therefore, higher SCS may be associated with greater sexual well-being for both partners in couples coping with FSIAD.

Although being motivated to be responsive to a partner’s sexual needs can be associated with greater sexual well-being for both partners, if the motivation to meet a partner’s sexual needs is extreme and excludes one’s own needs—termed high unmitigated sexual communion (USC]—this is no longer associated with greater sexual well-being and, instead, may be associated with poorer well-being [16,26]. Unmitigated communion differs from communal care in that it involves self-neglect [26], see also [27]. Thus, it is possible to be high in SCS without also being high in USC, as demonstrated in prior research [20,21]. Prior research has also demonstrated that whereas communion is associated with positive views of the self and others, and better interpersonal well-being and health, unmitigated communion is negatively associated with these factors [28]. Individuals high in unmitigated communion regularly neglect their own needs and well-being and are overly concerned with the needs of their partner, which takes the value of interpersonal connectedness to an unhealthy extreme [26]. In fact, when relationship stress was high, people higher in unmitigated communion experienced lower positive affect and higher levels of anxiety, depression, and negative affect [29].

In the sexual domain specifically, on days when people reported higher USC, they reported lower sexual and relationship satisfaction [21]. In addition, in a clinical sample of couples coping with the woman’s pain during sex, on days when women reported higher USC, they reported lower sexual satisfaction and sexual function, and both partners reported lower relationship satisfaction [20]. These findings suggest that sexual communal motivation that excludes one’s own needs might be associated with both partners’ lower sexual and relationship satisfaction for couples coping with sexual problems. In clinical cases of women coping with FSIAD, it is common for women to report accepting a partner’s sexual advances due to fears about losing the relationship, but then feeling dissatisfied with the sexual encounter [7]. Therefore, high unmitigated sexual communal may be associated with poorer sexual well-being for both partners.

### Sexual communal motivation and sexual goals

A person’s broader motivation to meet their partner’s sexual needs may also be associated with their specific goals for engaging in sex with their partner. That is, SCS and USC might be differentially associated with a person’s *reasons* for engaging in sex. Approach-avoidance motivational theory has been applied to sexuality and identifies two broad categories of goals for engaging in sex. *Approach sexual goals* involve engaging in sex in pursuit of positive outcomes, such as to promote intimacy or express love for a partner, whereas *avoidance sexual goals* involve engaging in sex to avert negative outcomes, such as to avoid conflict or the loss of a relationship [16,30,31]. In one daily experience study of long-term couples, those higher in SCS reported engaging in sex more for approach goals, but not for avoidance goals, and higher approach sexual goals are one reason why people higher in SCS reported higher daily sexual desire [16]. In contrast, people higher in USC tend to place greater attention on negative cues during sex, such as feeling bored or distracted, and less attention on positive sexual cues, such as their partner’s responsiveness [21]. Women with FSIAD seeking therapy commonly report lower approach goals for sex (i.e., to connect with their partner) and higher avoidance goals (i.e., to avoid losing their partner) [7]—which is consistent with studies comparing women with a sexual dysfunction to healthy controls [32]—and their goals may be differentially associated with their SCS and USC.

Among couples coping with FSIAD, one partner’s sexual communal motivation may also be associated with their partner’s sexual goals. Research has demonstrated that, among women with low sexual desire, partner-specific characteristics including whether a partner is motivated to meet her sexual needs or if she feels her partner has sexual needs that she cannot meet, are associated with the woman’s feelings of desire [33]. Research with community samples has found that on days when one partner is higher in SCS, the other partner focuses more on positive cues during sex, such as their connection with their partner and the partner’s responsiveness [21]. People higher in SCS are also perceived by their partner as more responsive during sex [19]. Therefore, among couples coping with FSIAD, it is possible that one partner’s SCS will be associated with either partner’s higher sexual approach goals. In contrast, previous work has shown no significant associations between SCS and avoidance goals for sex [16].

In prior research, having a partner higher in unmitigated sexual communion was not associated with a greater focus on either positive or negative cues during sex [21]. Given that people higher in unmitigated communion are overly concerned about meeting their partners’ needs [34], it is possible that, in the sexual domain, having a partner higher in USC is associated with a greater likelihood of engaging in sex to avoid upsetting them (i.e., for higher avoidance sexual goals). However, although people higher in unmitigated communion often perceive that their partner experiences more negative feelings about them such as annoyance or disappointment [35], it is not clear if these perceptions are accurate. In one study, some women with low sexual desire self-identified as self-sacrificing, martyr figures, having joyless sex driven solely by their partner’s needs [13]. However, these ostensibly self-sacrificial acts, when unmitigated by one’s own needs, may be motivated by a desire to avoid negative outcomes (e.g., conflict or losing the relationship) and, in turn, be associated with the very outcomes that the person wishes to avoid [36]. Therefore, among couples coping with FSIAD, it is possible that one partner’s USC will be associated with both partner’s higher sexual avoidance goals.

## Current study

In the current study, we recruited a sample of couples coping with FSIAD to investigate the role of SCS and USC in the sexual well-being (i.e., dyadic sexual desire, sexual satisfaction, sexual distress) and sexual goals (i.e., approach and avoidance sexual goals) of both women with FSIAD and their partners. We expected that when women or their partners were higher in SCS, both partners would report greater sexual well-being and stronger approach goals for sex, but that when women and partners were higher in USC both partners would report lower sexual well-being and stronger avoidance goals for sex. Previous research testing links between sexual communal motivation and well-being has been conducted with community couples who engaged in sex once a week or more, on average (e.g., [16]) or specifically on days when couples engaged in sex (e.g., [20, 36, 37]). In fact, people higher in SCS are more likely to engage in sex even when their desire is low [18], and sexual frequency is associated with relationship and sexual satisfaction [38,39]. But, many women coping with FSIAD avoid sex with their partner [7,40]. It is possible that in couples coping with FSIAD, the associations between SCS and USC and sexual well-being might be driven by how frequently the couple reports engaging in sex. Therefore, we conducted additional, exploratory tests of whether any associations were moderated by how frequently the couple engaged in sex. Given that very little is known about evidence-based targets for intervention in the treatment of FSIAD and no studies have focused on the interpersonal factors that are associated with the well-being of both members of couples coping with FSIAD, the current study will provide novel insight into factors that might protect couples coping with chronic low desire from lower sexual well-being.

## Materials and methods

### Participants

To be eligible for the study individuals had to be 18 years or older, and both partners had to agree to participate. Additional eligibility criteria included couples being in a committed relationship for a minimum of six months, having previous sexual experience, seeing each other in person at least four times each week, having English language fluency, and not currently undergoing hormonal therapy (hormonal contraceptives were allowed). We were interested in recruiting established couples coping with FSIAD, therefore required a minimum relationship length of six months. In addition, we were interested in the sexual experiences of couples coping with FSIAD, and therefore recruited couples who were geographically close to each other and saw each other regularly so they would have the opportunity to engage in sexual activity.

A total of 215 women completed a brief telephone screening conducted by a research assistant to determine preliminary eligibility, and 174 of these women met the initial eligibility criteria to continue to the clinical interview. The telephone screen included verification of inclusion and exclusion criteria, initial verification of FSIAD symptoms (but not a complete clinical assessment), and confirmation that their partner was willing to participate in the study. The most common reason for exclusion based on the brief telephone screening was that the woman did not meet their criteria of having persistent low desire accompanied by distress (meaning it was extremely unlikely they would meet the diagnostic criteria for FSIAD). Of these 174 women, 31 women were no longer interested in participating, which left 143 women who then completed the clinical interview. Women underwent a psychodiagnostic semi-structured telephone clinical interview conducted by either a clinical psychologist or a senior PhD student in Clinical Psychology (supervised by a clinical psychologist) to determine if they met diagnostic criteria for FSIAD. Of these, 25 women did not meet the criteria for FSIAD, following the psychodiagnostic clinical interview. The remaining 21 exclusions were due to one or both partners not completing the survey within the four-week allotted time (*n* = 6) or failing attention checks embedded in the survey (*n* = 15).

Our final sample included 97 women with FSIAD and their partners (*Ns* = 88 men, 6 women, 3 other) recruited from both online (from Kijiji, Facebook, Twitter and Reddit) and physical advertisements (in hospitals, universities, and community buildings) from September 2016 to May 2018 across North America. Only 1.0% of data were missing for partners’ sexual orientation. Table 1 provides complete participant demographics.

**Table 1:** Sample demographics (N = 97 couples).

	Women		Partners	
Characteristic	*M* (range) or *n*	*SD* or %	*M* (range) or *n*	*SD* or %
Age (years)	31.03	7.73	32.25	9.27
	(19.07–57.48)		(19.07–70.34)	
Ethnicity				
African American/Black	2	2.1%	2	2.1%
Asian American/Asian	9	9.4%	9	9.4%
Caucasian/White	69	71.9%	74	77.1%
East Indian	1	1%	1	1%
Hispanic/Latino/Latina	4	4.2%	2	2.1%
Middle Eastern/Central Asian	3	3.1%	3	3.1%
Biracial/Multiracial	3	3.1%	3	3.1%
Other	5	5.2%	2	2.1%
Annual income (household; CAD$)				
$0–19,999	13	13.6%	-	-
$20,000–39,999	16	16.7%	-	-
$40,000–59,999	15	15.7%	-	-
$60,000–79,999	20	20.8%	-	-
$80,000–99,999	11	11.5%	-	-
≥ $100,000	21	21.9%	-	-
Relationship status				
Dating	10	10.3%	-	-
Cohabitating	26	26.8%	-	-
Common-law	13	13.4%	-	-
Engaged	7	7.2%	-	-
Married	41	42.3%	-	-
Self-identified sexual orientation				
Straight/Heterosexual	68	70.1%	82	84.5%
Bisexual	15	15.5%	6	6.2%
Queer	4	4.1%	2	2.1%
Pansexual	4	4.1%	-	-
Lesbian	3	3.1%	3	3.1%
Asexual	1	1.0%	3	3.1%
Other	2	2.1%	-	-
Relationship duration (months)	92.03	85.25	-	-
	(7.5–419)			
FSIAD duration (months)	54.65	63.14	-	-
	(3–372)			
Study variables				
Sexual communal strength	2.36	.65	3.13	0.51
	(0.50–3.67)		(1.83–4.0)	
Unmitigated sexual communion	2.53	.78	3.62	0.66
	(1–4.33)		(1.67–5.0)	
Approach sexual goals	5.47	1.22	6.29	0.80
	(1.67–7.0)		(2.67–7.0)	
Avoidance sexual goals	4.14	1.50	3.14	1.64
	(1–7)		(1–7)	
Dyadic sexual desire	17.64	9.05	39.57	8.22
	(0 ‒ 43)		(6–54)	
Sexual satisfaction	20.98	5.48	23.80	6.22
	(5–35)		(10–35)	
Sexual distress	30.08	9.85	17.66	10.35
	(7–50)		(0–50)	

*Note*. FSIAD duration was based on self-report and separate from the inclusion assessment.

Using the Actor Partner Interdependence Model (APIM) power Shiny app [41] and associations between SCS and relationship satisfaction from previous cross-sectional research with community couples [19] where the actor effect = .32 and the partner effect = .24, we had 95% power to detect our effects in the current sample. That is, based on these estimated effect sizes, a sample of 93 couples was needed to detect the effects at 95% power.

### Procedure

Participants who were eligible for the study were screened and diagnosed through a telephone clinical interview by a clinical psychologist to confirm FSIAD. This clinical interview was developed based on prior studies’ models [9,42] and refined based on our teams’ clinical expertise. The research assistant then spoke briefly to the other partner in the relationship to confirm their interest in participating in the study. Couples who decided to participate in the study received an individualized link to the online consent form and once consent was provided, participants were then directed to the online survey. Qualtrics online survey software was used to distribute the surveys. Members of each couple were required to complete the survey within four weeks and were instructed to do so separately and without discussing their responses with each other. As part of the follow-up protocol, a series of reminders to complete the survey was sent out to participants. After completing the survey, participants received online resources for sexuality and relationships. Once both members of the couple completed the survey, they were each compensated with an $18 CAD gift card to Amazon.com/ca. The studies were approved by the authors’ institutional research ethics boards.

The current data were collected as part of a larger study investigating interpersonal factors that are associated with the sexual, psychological, and relationship well-being of couples coping with FSIAD. The study was advertised as a study of women with low sexual desire and their partners. One of our key goals is tested in the current paper—the role of SCS and USC in women and partners’ sexual well-being. Some data from the larger study have also been published in which we compared this sample of couples coping with FSIAD to a control sample on measures of personal, relational and sexual well-being [10]. See also Rosen et al. [10] for full sample and procedural details.

#### Measures

All questionnaires can be found in the Supporting Information (S1 File).

**Socio-demographics:** Participants reported their ethnicity, gender, sexual orientation, and age. Women also reported their relationship status and duration of FSIAD (see S1 File).

**Sexual communal strength:** SCS was measured with six items that were previously adapted from a general measure of communal strength [43]. The measure of SCS has been used in previous research (e.g., [16]) and has been shown to be a valid and reliable measure of the motivation to be communally responsiveness to a partner’s sexual needs (for more information see S1 File). Respondents indicate their extent of agreement with each item (e.g., “How happy do you feel when satisfying your partner’s sexual needs”) on a scale from 0 (*not at all*) to 4 (*extremely*). Scores on this scale are averaged and can range from zero to four, with higher scores indicating greater motivation to meet their partner’s sexual needs (FSIAD women α = .73; partners α = .67).

**Unmitigated sexual communion:** To measure the extent to which participants focus on their partner’s sexual needs to the exclusion of themselves, six items were previously adapted from a validated measure of unmitigated communion [20,37,44]. For additional information about the reliability and validity of this measure, see S1 File. Example items include: “It is impossible for me to satisfy my own sexual needs if they interfere with the needs of my partner,” and “I always place my partner’s sexual needs above my own.” Items were rated on a five-point scale from 1 (*strongly disagree*) to 5 (*strongly agree*). Scores on this scale are averaged and can range from one to five, with higher scores indicating higher prioritization of a partner's sexual needs in neglect of one's own needs (FSIAD women α = .76; partners α = .66).

**Approach and avoidance sexual goals:** Sexual goals were assessed with a 12-item measure used in previous research [30,45]. A version of this measure—the Sexual Motivations Scale-Revised—was originally validated by Cooper, Shapiro and Powers [43]. The current version is a truncated version with only the two subscale factors relevant to the context of romantic relationships. Participants rated the importance of six approach (e.g., “to promote intimacy in my relationship”) and six avoidance (e.g., “to prevent my partner from falling out of love with me”) interpersonal goals in influencing their decision to engage in sex on seven-point scales ranging from 1 (*not at all important*) to 7 (*extremely important*). The mean is calculated for each subscale. Higher approach sexual goal scores indicate stronger goals toward potential positive outcomes, while higher avoidance sexual goal scores indicate stronger goals away from potential negative outcomes, (FSIAD women approach goals α = .86; partners α = .83; FSIAD women approach goals α = .84; partners α = .91).

**Sexual desire:** Dyadic sexual desire for participants’ own partners was assessed with the seven items of the partner-focused dyadic sexual desire subscale from the 14-item Sexual Desire Inventory (SDI-2; [46]), as per Moyano, Vallejo-Media, and Sierra’s [47] recommendation. Items are rated from 0 (*no desire*) to 8 (*strong desire*). Example items include: “When you have sexual thoughts, how strong is your desire to engage in sexual behaviour with a partner?” and “During the last month, how often have you had sexual thoughts involving your partner?” Scores on this subscale are summed and can range from 0 to 56, with higher scores indicating higher levels of dyadic sexual desire for one’s partner, (FSIAD women sexual desire α = .79; partners α = .85).

**Sexual satisfaction:** Overall sexual satisfaction was assessed with the Global Measure of Sexual Satisfaction scale (GMSEX) [48]. Participants are asked to describe their overall sexual relationship with their partner in five bipolar dimensions (i.e., very bad/good, unpleasant/pleasant, negative/positive, satisfying/unsatisfying, and worthless/valuable) on a 7-point scale ranging from 1 to 7. Ratings are summed, and total scores can range from five to 35, with higher scores indicating greater sexual satisfaction, (FSIAD women sexual satisfaction α = .87; partners α = .92).

**Sexual distress:** Sexual distress was assessed with the 13-item Female Sexual Distress Scale-Revised (FSDS-R) [49]. Participants rated on a 5-point Likert-type scale how frequently they experienced distress (e.g., stress or guilt) about their sex lives. Intensity of distress is rated from 0 (*not at all*) to 4 (*extremely*). Ratings are summed, and total scores can range from 0 to 52, with higher scores indicating higher sexual distress. Although the FSDS-R was originally developed specifically for women, it uses gender-neutral language and has been validated in men [50], (FSIAD women sexual distress α = .91; partners α = .92).

**Sexual intercourse frequency:** Sexual intercourse frequency was measured with one item: “During the past 4 weeks, how often did you and your partner engage in sexual intercourse with vaginal penetration?” Response options were 0 (not at all), 1 (once or twice), 2 (once a week), 3 (2–3 times a week), 4 (4–5 times a week), 5 (once a day), or 6 (more than once a day).

### Data analyses

Data were analyzed with multilevel modeling using mixed models in SPSS Version 23.0 where partners were nested within couples to account for the non-independence of couple data [51]. Analyses were guided by the Actor Partner Interdependence Model. All models included women and their partners’ SCS and USC as predictors. We ran separate models for each outcome (five models in total for the main analyses). In the analyses, we assessed the associations between women’s and partners’ SCS and USC and their own outcomes (i.e., actor effects) and the associations between women’s and partner’s SCS and USC and their partner’s outcomes (i.e., partner effects). The coefficients reported are unstandardized betas, interpreted as the change in the outcome for every one-unit increase in the predictor. These coefficients act as indications of the size of the effect. Correlations among all study variables are reported in Table 2.

**Table 2:** Correlations among all study variables.

Variable	1	2	3	4	5	6	7
1. Sexual Communal Strength	**-.14**	.63**	.39**	.10	-.02	.31**	.09
2. Unmitigated Sexual Communion	.55**	**.09**	.25*	.25*	-.08	.08	.12
3. Approach Sexual Goals	.31**	.31**	**.14**	.03	.12	.28**	.20*
4. Avoidance Sexual Goals	.01	.16	.14	**.14**	-.08	-.14	.34**
5. Sexual Desire	.14	.04	.04	-.05	-.64**	.17	-.24*
6. Sexual Satisfaction	.10	-.01	.07	.00	.21*	.42**	-.35**
7. Sexual Distress	-.05	.18	.14	.06	-.12	-.63**	**.08**

*Note*. Correlations are among all study variables. Women’s correlations are above the diagonal; partner’s correlations are below the diagonal; bolded correlations are between women and partner reports.

* *p* < .05.

**p < .01.

## Results

### Associations between sexual communal motivation and sexual well-being

First, we tested associations between women with FSIAD and their partner’s sexual communal motivation (SCS and USC) and both partners’ sexual well-being (i.e., sexual desire, sexual satisfaction, sexual distress). Consistent with predictions and reported in Table 3, when women with FSIAD reported higher SCS, they reported higher sexual desire for their partner (*p* = .042), and when partners reported higher SCS, partners also reported higher sexual desire (*p* = .003). However, there were no significant associations between USC and sexual desire. In addition, and as predicted, when women with FSIAD reported higher SCS, both women (*p* = .001) and their partners reported greater sexual satisfaction (*p* = .01; see Table 3). Contrary to predictions, when partners reported higher SCS, neither women with FSIAD nor their partners reported feeling more sexually satisfied. When women with FSIAD or their partners reported higher USC, there were no significant associations with sexual satisfaction.

**Table 3:** Associations between sexual communal strength and unmitigated sexual communion and sexual well-being.

	W’s sexual desire		P’s sexual desire		W’s sexual satisfaction		P’s sexual satisfaction		W’s sexual distress		P’s sexual distress	
	*b* (SE)	*t*	*b* (SE)	*t*	*b* (SE)	*t*	*b* (SE)	*t*	*b* (SE)	*t*	*b* (SE)	*t*
W’s SCS	3.73 (1.81)	2.06*	2.13 (1.59)	1.34	3.68 (1.08)	3.41***	3.29 (1.25)	2.64**	1.61 (2.01)	.80	-2.99 (2.15)	-1.39
P’s SCS	-3.95 (2.11)	-1.87	5.63 (1.85)	3.04*	2.05 (1.25)	1.63	2.72 (1.45)	1.87	.45 (2.34)	.19	-5.33 (2.47)	-2.16*
W’s USC	-.11 (1.51)	-.07	-2.07 (1.32)	-1.57	-1.17 (.89)	-1.31	.11 (1.03)	.11	.02 (1.67)	.02	-.11 (1.82)	-.06
P’s USC	-.42 (1.60)	-.26	1.75 (1.40)	1.25	-.90 (.95)	-.95	-1.20 (1.10)	-1.09	2.91 (1.77)	1.64	5.08 (1.89)	2.69**

*Note*. W = women; P = partner; SE = standard error; SCS = sexual communal strength; USC = unmitigated sexual communion.

* *p* < .05.

** *p* < .01.

*** *p* < .001.

We also tested sexual distress, a negative indicator of sexual well-being. When partners reported higher SCS, they reported lower sexual distress (*p* = .034), but when partners reported higher USC, they reported higher sexual distress (*p* = .009). However, there were no associations between partner’s sexual communal motivation and women’s sexual distress or between women’s sexual communal motivation and their own or their partner’s sexual distress.

### Associations between sexual communal motivation and sexual goals

Next, we tested associations between women with FSIAD and their partner’s sexual communal motivation (SCS and USC) and both partners’ sexual goals. When women with FSIAD reported higher SCS, both they (*p* = .005) and their partners (*p* = .037) reported having sex more for approach goals (see Table 4). In addition, when partners reported higher SCS, they reported having sex more for approach goals (*p* = .047), but there was no association with women’s approach goals. As expected, there were no significant associations between SCS and avoidance sexual goals for either partner. There were also no significant associations between USC and approach or avoidance sexual goals for either partner.

**Table 4:** Associations between sexual communal strength and unmitigated sexual communion and sexual goals.

	W’s approach sexual goals		P’s approach sexual goals		W’s avoidance sexual goals		P’s avoidance sexual goals	
**Predictors**	*b* (SE)	*t*	*b* (SE)	*t*	*b* (SE)	*t*	*b* (SE)	*t*
W’s SCS	.71 (.24)	2.91**	.33 (.16)	2.11*	-.04 (.31)	-.14	-.26 (.35)	-.77
P’s SCS	-.00 (.28)	-.01	.37 (.18)	2.01*	.43 (.36)	1.20	-.42 (.40)	-1.05
W’s USC	.04 (.20)	.18	-.25 (.13)	-1.89	.45 (.26)	1.75	.20 (.29)	.71
P’s USC	-.07 (.21)	-.34	.26 (.14)	1.86	-.02 (.27)	-.07	.54 (.30)	1.79

*Note*. W = women; P = partner; SE = standard error; SCS = sexual communal strength; USC = unmitigated sexual communion.

* *p* < .05.

** *p* < .01.

*** *p* < .001.

We also ran all analyses reported above with age and relationship duration controlled. With two exception, all of the effects remain significant. The exceptions were that the association between men’s SCS and approach goals and the association between women with FSIAD’s SCS and desire become marginal when relationship duration is controlled (*p* = .053 and 0.066, respectively).

#### Correction for multiple tests

Given the multiple tests conducted in this study, using a false discovery rate (FDR) of 15%, we applied the Benjamini, Krieger, and Yekutieli (BKY) adaptive linear step-up procedure [52] to our findings. This method reduces risk of Type 1 error by using the *p*-value distribution to calculate adjusted alphas for each significant test. Five of the above-reported associations between sexual communal motivation and sexual well-being remained significant using this procedure. Women’s SCS remained positively associated with their own sexual satisfaction, their own sexual approach goals, and their partner’s sexual satisfaction. Partner’s SCS remained positively associated with their own sexual desire, and partner’s USC remained positively associated with their own sexual distress. However, four effects were not retained when controlling for an FDR of .15, meaning there is a greater likelihood of these being false positives and they should be interpreted with caution. These effects include the associations between women’s SCS and their own desire and their partner’s approach goals, and the association between a partner’s SCS and their own distress and sexual approach goals.

#### Exploring differences by sexual intercourse frequency

In the next set of analyses, we conducted exploratory tests of whether the associations between sexual communal motivation and sexual well-being are moderated by sexual intercourse frequency. Previous research has shown that women with FSIAD may avoid circumstances in which sexual activity is likely to occur and engage in sexual avoidance behaviour with their partner [7,40]. In fact, in the current sample, about a quarter (23.7%) of the couples did not engage in sexual intercourse in the past four weeks. The average sexual intercourse frequency was about once or twice in the past four weeks. Our measures of SCS and USC are focused on meeting a partner’s sexual needs, which might be more relevant when couples are engaging in regular sexual activity. Therefore, in a series of exploratory analyses, we tested whether the effects differed for couples who engage in more frequent intercourse compared to couples who report infrequent intercourse. The multiple testing correction was not applied to the sexual frequency moderations as these are exploratory analyses and the correction is meant for predicted effects. Only one of the significant effects reported above was moderated by sexual intercourse frequency; frequency of intercourse significantly moderated the association between partners’ SCS and their own approach sexual goals, *b* = 0.33, *SE* = 0.16, *t*(87.09) = 2.11, *p* = .038, 95% CI (0.019, 0.65). Follow-up simple effects tested at +/-1 standard deviation revealed that, for couples who reported more frequent intercourse, partners higher in SCS reported higher approach goals for sex, *b* = 0.62, *SE* = 0.22, *t*(86.98) = 2.85, *p* = .005, 95% CI (0.19, 1.05). However, when frequency of intercourse was low, there was no association between partners’ SCS and their approach goals, *b* = -0.05, *SE* = 0.26, *t*(87.39) = -0.17, *p* = .86, 95% CI (-0.57, 0.48). A number of additional moderations by sexual intercourse frequency emerged for effects that were not significant in the main analyses. Overall, these analyses revealed that additional associations between sexual communal motivation and sexual well-being and sexual goals are significant only for couples who engage in more frequent intercourse. Sexual intercourse frequency moderated the association between partners’ SCS and their own sexual satisfaction (*b* = 2.80, *SE* = 1.20, *t*(88.07) = 2.33, *p* = .022, 95% CI (0.42, 5.18). As shown in Table 5, simple effects tests revealed that when couples report having more frequent intercourse, partners’ SCS was associated with their own higher sexual satisfaction, but when sexual intercourse frequency was low, partners’ SCS was not associated with their sexual satisfaction.

**Table 5:** Simple effects of sexual communal motivation on own and partner’s sexual satisfaction (i.e., actor and partner effects) at low and high levels of sexual intercourse frequency.

	Own sexual satisfaction (i.e., actor effects)					
	*b*	*SE*	*t*	*df*	*p*	*95% CI*
Low sexual intercourse frequency						
P’s SCS	-0.15	1.99	-0.07	88.48	0.941	-4.09, 3.80
P’s USC	1.33	1.57	0.398	88.59	0.398	-1.79, 4.46
W’s USC	0.76	1.16	0.66	88.24	0.514	-1.54, 3.05
High sexual intercourse frequency						
P’s SCS	5.39*	1.65	3.27	87.70	0.002	2.11, 8.66
P’s USC	-3.25*	1.33	-2.45	88.20	0.016	-5.88, -0.61
W’s USC	-3.94*	1.43	-2.75	88.61	.007	-6.78, -1.10
	Partner sexual satisfaction (i.e., partner effects)					
Low sexual intercourse frequency						
P’s USC	-1.73	1.44	-1.20	88.62	.233	-4.60, 1.13
W’s USC	1.69	1.31	1.29	88.18	.200	-0.91, 4.30
High sexual intercourse frequency						
W’s USC	-3.43*	1.44	-2.39	88.36	.019	-6.29, -0.58

*Note*. W = women; P = partner; SE = standard error; SCS = sexual communal strength; USC = unmitigated sexual communion. Partner = whichever person is the partner *of* the person reporting USC. Low and high sexual intercourse frequency represent simple effects tests conducted at +/- 1 standard deviation of sexual intercourse frequency.

*p < 0.05.

Sexual intercourse frequency also significantly moderated the association between women’s USC and their own, *b* = -2.37, *SE* = 0.95, *t*(88.05) = -2.51, *p* = .014, 95% CI (-4.25, -0.49), and their partners’ sexual satisfaction, *b* = -2.59, *SE* = 0.95, *t*(88.34) = -2.73, *p* = .008, 95% CI (-4.48, -0.70), and partners’ USC and their own sexual satisfaction, *b* = -2.32, *SE* = 1.02, *t*(87.65) = -2.27, *p* = .026, 95% CI (-4.35, -0.29). Simple effects tests revealed that, when couples reported having more frequent intercourse, women’s higher USC was associated with their own lower sexual satisfaction as well as their partner’s lower sexual satisfaction (see Table 5). When couples reported having more frequent intercourse, partner’s USC was associated with their own lower sexual satisfaction (see Table 5). However, when couples reported having less frequent intercourse, women’s USC was not associated with their own sexual satisfaction or their partner’s sexual satisfaction (see Table 5). When couples reported having less frequent intercourse, partner’s USC was also not associated with their own sexual satisfaction (see Table 5) or women with FSIAD’s sexual satisfaction (see Table 5). In sum, when couples reported having more (but not less) frequent intercourse, women’s higher USC was associated with both partners’ lower sexual satisfaction, and partner’s higher USC was associated with their own lower sexual satisfaction.

Given that the question about sexual frequency was limited to intercourse (and was not inclusive of all sexual activity) and may be interpreted differently based on gender and sexual orientation, we re-ran all moderations by sexual intercourse frequency with only the mixed-sex cis-gender couples. That is, we removed eight couples where partners identified as a woman or a trans person or either partner selected ‘other’ for their gender. In the remaining sample of mixed-sex cis-gender couples (*N* = 89), all of the significant moderations by sexual intercourse frequency remained significant.

## Discussion

The current research adds to a growing body of literature highlighting the role of interpersonal factors in how women and couples cope with a sexual dysfunction [10,20,32,34,39,53–56]. In the current study, we demonstrate that being communally motivated to meet a partner’s sexual needs was associated with greater sexual well-being in a sample of couples coping with FSIAD. When women coping with FSIAD were higher in SCS they reported having sex more for approach goals and both they and their partner report higher sexual satisfaction. Partners who were higher in SCS also reported higher sexual desire and sexual satisfaction (although the association between partner’s higher SCS and their own sexual satisfaction was only retained for couples who engaged in more frequent intercourse). We also found preliminary evidence that when women with FSIAD report higher SCS, they also report higher sexual desire and their partner report higher approach sexual goals, and when partners reported higher in SCS, they report lower distress and higher approach goals. However, although consistent with theory and prior research with community samples [16] and other populations of couples coping with sexual problems [20,37], these effects were not retained with the multiple comparison correction, suggesting that there is a greater chance of these effects being false positives and more evidence is needed.

When the motivation to meet a partner’s sexual needs is to the exclusion of a person’s own needs—higher USC—women and partners no longer reported greater sexual well-being, and in some cases, USC was associated with poorer well-being, particularly among couples reporting more frequent intercourse. That is, when partners of women with FSIAD reported higher USC they reported higher sexual distress. And, for couples who reported engaging in more frequent intercourse (approximately once a week or more), when women were higher in USC, both women and partners reported lower sexual satisfaction, and when partners were higher in USC, partners felt less sexually satisfied. Higher USC was not associated with lower sexual well-being for couples engaging in less frequent intercourse.

Overall, the effects found in the current study between SCS and sexual well-being were small to moderate. Although a woman's SCS was associated with her partner's sexual satisfaction and sexual approach goals, most of the significant effects are primarily actor (as opposed to partner) effects—that is, associations between a person's own sexual communal motivation and their own sexual well-being. In addition, when entered together as predictors, SCS is more strongly associated with sexual well-being than USC. After accounting for SCS, most of the associations between USC and sexual well-being were not significant.

### Sexual communal strength

The current findings are consistent with past research on the positive associations of SCS with sexual well-being for both community [19,21] and clinical [20,37] samples of couples. Past research has found that people higher in SCS are more likely to maintain desire over time, even in a sample of long-term couples where desire tends to decline [16]. In past work with community couples, when a person’s own desire was lower than their partner’s desire, people higher in SCS tended to focus more on the benefits of having sex for their partner and their relationship and less on the costs of engaging in sex, and in turn, they were more likely to engage in sex in these situations and both partners report greater sexual satisfaction as a result [18]. It is possible that, even though couples in the current sample are coping with low desire, SCS helps them focus on the positive aspects of sex (e.g., intimacy, physical pleasure for self and partner)—as is encouraged in psychosocial treatments of low sexual desire [57]—and, in turn, they may be more open to sexual activity. In the current sample, when partners of women coping with FSIAD were higher in SCS, they also reported higher sexual desire, which is consistent with finding in community samples of long-term couples [16,31], and suggests that being motivated to meet a partner’s sexual needs is also linked to one’s own sexual well-being. In the current sample, women coping with FSIAD who reported higher SCS also reported higher sexual desire, although this finding was not retained with a multiple comparison correction.

We found that SCS was associated with feeling more sexually satisfied; when women with FSIAD were higher in SCS, both they and their partner reported higher sexual satisfaction. This finding is consistent with past research with community couples in which people higher in SCS were more likely to engage in sex when their desire was low (but their partner’s desire was high), and both partners reported feeling more sexually satisfied [58]. In the current study, when couples coping with FSIAD had more (as opposed to less) frequent intercourse, partners’ SCS was associated with them feeling more sexually satisfied. In previous research, the partners of individuals higher in SCS indicated that their partner was more responsive to their needs during sex, and perceptions of partner responsiveness was one main reason why they reported greater satisfaction [19]. Therefore, it is possible that women with FSIAD higher in SCS have partners who report greater sexual satisfaction because they perceive their FSIAD partner as more responsive. Future research is needed to test this possibility.

Consistent with previous research [16], when women with FSIAD were higher in SCS, they were more likely to engage in sex for approach goals, such as to enhance intimacy in their relationship. Previous work in community couples [16] has found that having stronger approach goals for sex is one reason why people higher in SCS report higher sexual desire. Therefore, having sex more for approach goals might be one path through which women with FSIAD who are high in SCS experience higher sexual desire. In our exploratory analysis, we found that when partners were higher in SCS, they were more likely to have sex for approach goals, but this was only among couples who reported more frequent intercourse. Perhaps if sex is infrequent, higher SCS does not translate into higher approach goals, or perhaps measures of sexual motivation are more difficult to complete when sexual frequency is low. There was no association between SCS and avoidance goals, suggesting that when couples coping with FSIAD are communally motivated to meet their partner’s sexual needs, they do not do so to avoid negative consequences, such as conflict or a partner’s disappointment. Instead, it seems that SCS is associated with women with FSIAD being more oriented towards the positive aspects of the sexual experience, consistent with research with community couples [21]. For women coping with FSIAD, being higher in SCS and having higher approach goals might mean adapting the couple’s sexual repertoire to accommodate the women with FSIAD’s low interest/arousal (e.g., engaging in activities that are more stimulating for the woman with FSIAD), which may be associated with higher levels of desire and arousal. In fact, one model of women’s sexual response patterns—the intimacy-based circular model of women’s sexual response [59]—proposes that emotional intimacy can motivate a woman to be more open to a sexual encounter (i.e., she may be motivated to engage in sex to experience emotional intimacy, akin to approach sexual goals), and in turn, she experiences more sexual arousal and desire, and, ultimately, sexual satisfaction.

### Unmitigated sexual communion

Although meeting a partner’s sexual needs was linked to benefits for couples coping with FSIAD, if the motivation to meet a partner’s sexual needs was extreme and did not take into account the person’s own needs, couples did not report greater sexual well-being, and, at times, USC was linked with lower sexual well-being. That is, when women were higher in USC, there were no significant associations with their own or their partner’s sexual well-being and when partners were higher in USC, partners reported more sexual distress. Similarly, in a daily experience study of couples coping with another female sexual dysfunction, on days when women were higher in USC, her partner did not report greater sexual well-being and instead reported being less satisfied with the relationship, and when partners were higher in USC, they reported poorer sexual function [including lower desire for sex; 20]. Previous research on unmitigated communion more broadly has also shown that although people higher in unmitigated communion are overly focused on meeting their partner’s needs, they may be more concerned about being the one to provide care to their partners than whether or not their partner’s needs are actually being met [26,34]. When partners of women with FSIAD are higher in USC, they might be focused on “fixing” the women’s low desire without aiming to understand her true feelings and interests, resulting in more negative emotions surrounding the sexual relationship. Alternatively, it could be that partners higher in USC feel they are not meeting their partner’s sexual needs (since she has low desire) and this experience is distressing.

In an exploratory analysis, we found that some associations between USC and sexual well-being were only significant for couples who reported engaging in more frequent sexual intercourse. That is, among couples who report more (compared to less) frequent intercourse, women’s higher USC was associated with their own and their partner’s lower sexual satisfaction. In research on general unmitigated communion, whereas people lower in unmitigated communion reported higher well-being when providing support to their partners, people higher in unmitigated communion did not experience greater well-being during support provision [60]. Findings such as these suggest that unmitigated communion is not associated with greater personal well-being when providing care to close others. People higher in unmitigated communion often have trouble asserting their own needs, which is related to lower well-being [26,35]. Therefore, women with FSIAD who are higher in USC may have trouble communicating their sexual needs to their partners and may resign to engage in sex based on their partner’s desires. When a woman with FSIAD is solely focused on meeting her partner’s sexual needs, neglects her own needs, and acquiesces to having more frequent sex, this may negatively impact both partners. Previous research shows that pressure to conform to conventional feminine ideals—such as a willingness to have sex as well as being perceptive to and being able to satisfy a partner’s sexual needs—are more pronounced in women who are coping with sexual problems [61]. Women with FSIAD higher in USC who have more frequent intercourse may be feeling pressure to focus on their partner’s sexual needs while devaluing their own needs, which is associated with lower sexual satisfaction for both partners. In addition, consistent with past work in community samples [21], when partners were higher in USC, they also reported lower sexual satisfaction. These findings suggest that, for partners of women with FSIAD, engaging in sex is most consequential (and negatively associated with partners’ sexual satisfaction) when they are higher in USC. Although partners higher in USC are solely focused on meeting their partner’s needs, women with FSIAD do not report greater sexual well-being associated with this focus, and their partners report lower sexual satisfaction. Future research is needed to further explore these possibilities.

Finally, although there is some evidence that people higher in USC focus on negative cues during sex [21], in the current study, we did not find that people higher in USC had stronger avoidance sexual goals, such as having sex to avoid conflict or their partner’s disappointment. In fact, USC was not significantly associated with approach or avoidance sexual goals. Since, in the current study, sexual goals were partner-focused, it is possible that people high in USC are more motivated to meet their partner’s needs as a way to regulate their own anxiety (and not to pursue positive or avoid negative relational outcomes). People high in unmitigated communion generally aim to provide care to close others as a way to restore their own self-esteem [34], and it is possible that applied to sexuality, this means that their reasons for engaging in sex might be more focused on regulating their own emotions. Indeed, in one study, among women coping with coital pain, their reason for engaging in sex with their partner included to restore their own image of themselves as a “real woman” or good relationship partner and to mitigate their own feelings of guilt [61].

### Strengths and limitations

The current study has several strengths. It established the importance of two novel interpersonal factors—SCS and USC—for the sexual well-being and sexual goals of couples coping with FSIAD and included the perspectives of both partners. Much of the previous research on women with clinically low desire has not included partners or considered a dyadic perspective [1,4,13,62], even though both partners are often included in psychotherapy for sexual dysfunctions, such as FSIAD [56,63]. To our knowledge, there are currently no empirically-supported couple-based treatment studies for FSIAD [56]; the lack of studies on interpersonal factors means that which factors to target in couples therapy have not been empirically based [63].

The current study also has limitations. The study is correlational and cannot confirm the causal direction of the effects. However, our theorized direction of effects is in line with theory and past research, including an experimental study in which enhancing people’s focus on their partner’s sexual needs (i.e., high sexual communal strength) led them to expect higher sexual and relationship satisfaction in an imagined situation of desire discrepancy with their partner [16,18,64]. Our study is also limited in that asking about sexual intercourse may not be relevant for some couples and is not inclusive of all partnered sexual activity.

It is also possible that the associations are bidirectional in FSIAD, where sexual well-being leads to SCS and USC. In addition, while we postulated about possible mechanisms, such as focusing on positive aspects of sex as mediating links between SCS and higher sexual desire and satisfaction, given the cross-sectional nature of our data, we did not test these mechanisms in the current research. Future longitudinal research following couples coping with FSIAD over time could help clarify the direction and mechanisms of the effects and test whether sexual communal motivation is linked to changes in sexual well-being and goals over time. Finally, the internal consistency of the measures of SCS and USC—while acceptable—were lower for partners than women with FSIAD. It is possible that it is more difficult to complete or interpret measures about meeting your partner’s sexual needs or that meeting a partner’s sexual needs has a different meaning when your partner has FSIAD. For example, one of the items on the SCS measure asks, “How high a priority for you is meeting the sexual needs of your partner?” For people with a partner who has FSIAD, it might be a high priority for them to be able to meet their partner’s sexual needs, but since their partner’s need might be to *not* engage in sex or their partner may express fewer sexual needs, this question might have a different meaning. Thus, future work might consider assessing the motivation to meet a partner’s sexual needs when their partner’s need is to *not* engage in sex, as has been assessed in couples transitioning to parenthood [24]. In samples of couples coping with a sexual dysfunction, it might be more important to examine how a person responds to the affected women’s disinterest in sex as opposed to their sexual needs.

## Conclusions

In sum, our results suggest that when couples coping with FSIAD report higher SCS, they also experience greater sexual satisfaction and desire and have intercourse more for approach goals, but when sexual communal motivation is not mitigated by the person’s own agency (high unmitigated sexual communion), this is not associated with greater sexual well-being and instead is associated with higher sexual distress and lower sexual satisfaction (findings for sexual satisfaction were only for couples who engaged in more frequent intercourse). The results suggest that promoting SCS, while maintaining a focus on one’s own needs, might be a target for improving the sexual well-being of couples with FSIAD. The findings of the present study contribute to an emerging body of research on sexual dysfunction and sexual motivation [65], and point to novel interpersonal variables that could inform the development of empirically based interventions for couples coping with FSIAD.

## Supporting information

S1 File All questionnaires and information on sexual communal motivation measures. (DOCX)
