# Supplementary material for: Sexual communal motivation in couples coping with low sexual interest/arousal: Associations with sexual well-being and sexual goals
Source: PLoS One. 2019 Jul 17;14(7):e0219768. doi: 10.1371/journal.pone.0219768 (PMC6636740; doi:10.1371/journal.pone.0219768)
Supplement: S1 File — (DOCX) [file pone.0219768.s001.docx]

**S1 File. All questionnaires and information on sexual communal motivation measures.**

**Sociodemographics (18 items).**

1. How did you find out about this survey?

- Reddit
- Kijiji
- Twitter
- Poster/flyer
- Doctor’s office
- Facebook
- Newspaper
- Other (please specify) [text box]

2. What is your month and year of birth (MM/YYYY) _________/_________

3. What country do you currently live in?

- United States
- Canada

5. What city and state/province/territory do you currently live in?

City:______________ State/Province/Territory: ________________

6. Which best describes your ethnicity?

- Aboriginal/Native American/American Indian/Alaska Native/First Nations
- African American/Black
- Asian American/Asian
- Caucasian/White
- East Indian
- Hispanic/Latino/Latina
- Middle Eastern/Central Asian
- Native Hawaiian/Other Pacific Islander
- Biracial/Multiracial
- Other (specify if you wish) [text box]

7. What is the gender with which you identify?

- Male
- Female
- Trans-identify as male
- Trans-identify as female
- Other (specify if you wish) [text box]

8. What is your biological sex? (A measure in this survey asks questions that relate to your physical body/physiology. The question below is meant to determine which version of this measure is most appropriate for you to complete.)

- Female
- Male
- Intersex

9. [Use branching so only women get this measure] How do you characterize your menopausal status?

- Pre-menopausal (you have not yet started menopause; you are still menstruating with no symptoms of menopause)
- Peri-menopausal (you have started to experience symptoms of menopause such as irregular periods, but are still menstruating periodically)
- Post-menopause (you have completed menopause; menstruation has fully stopped)
- I’m not sure

10. How many years of schooling do you have (starting from first grade)?

| [Dropdown menu ranging from 1 to 30+] |
| --- |

11. What is the approximate total annual income of your household? (i.e., the total income for you and any partner you live with before taxes)

- $0 - $9,999
- $10,000 - $19,999
- $20,000 - $29,999
- $30,000 - $39,999
- $40,000 - $49,999
- $50,000 - $59,999
- $60,000 - $69,999
- $70,000 - $79,999
- $80,000 - $89,999
- $90,000 - $99,999
- $100,000 and over

12. What is your sexual orientation?

- Asexual
- Bisexual
- Gay
- Lesbian
- Straight/Heterosexual
- Pansexual
- Queer
- Other (specify if you wish) [text box]

13. Which of the following best describes your relationship with your romantic partner?

- Dating
- Living together (not common-law or married)
- Common-law
- Married
- Engaged
- Other

14. How long have you been in a relationship with your partner? Please count from the start of the relationship. For example, if you dated for 2 years and have been married for 2 years and 3 months you should answer 4 years and 3 months.

______ Years _______Months

15. Do you have any children? ☐ No ☐ Yes – Ages:

Next we need to ask you a few questions about your medical history.

16. Do you have any chronic or ongoing diseases or illnesses, such as liver or cardiac disease, or neurological disease?

17. Are you currently taking any medications?

☐ No ☐ Yes *If Yes:* Please report what they are and their doses

18. Have you been diagnosed with any psychiatric disorder, such as depression or an anxiety disorder or an eating disorder? ☐ No ☐ Yes

If yes: please indicate what disorder _________________

How long have you been experiencing problems with low sexual interest and/or arousal? (Compute total in months): ________

Have you experienced these problems only with your current partner, or also with past partners? ____________________

**Measure of Sexual Communal Strength.**

In our previous research, we have demonstrated the reliability and validity of the measure of sexual communal strength (SCS). Across diverse samples, including long-term couples (Day et al., 2015; Muise et al., 2013; Muise & Impett, 2015), new parent couples (Muise, Rosen, Kim, & Impett, 2017), a sample of couples coping with a sexual dysfunction (Muise, Bergeron, Impett, & Rosen, 2017), and a sample of individuals who are in consensually nonmonogamous (CNM) relationships (Muise, Laughton, Moors & Impett, 2018), the measure demonstrated adequate reliability, with Cronbach’s alphas ranging from .70 to .88.

Sexual communal strength is highly correlated with general communal strength (*r* = .59, *p* < .001; Muise et al., 2013), demonstrating convergent validity. Additionally, sexual communal strength is associated with both partners’ relationship quality above and beyond their motivation to meet their partner’s more general needs (Muise & Impett, 2015), indicating that sexual communal strength is a separate construct than general communal strength. As evidence of construct validity, people higher in SCS are perceived by their partners as more responsive to their needs during sex (Muise & Impett, 2015), suggesting that a person’s level of SCS is detected by their romantic partner. The predictive validity of the SCS measure is demonstrated in one study where people higher in SCS were more likely, over the course of a 21-day daily experience study, to engage in sex with their partner on days when their partner was interested in sex, but their own personal desire for sex was low (Day et al., 2015).

**Sexual Communal Strength (6 items).**

*Instructions*: Keeping YOUR ROMANTIC PARTNER in mind, answer the following questions. Please rate each item from 0 = not at all to 4 = extremely.

1. How far would you be willing to go to meet your partner's sexual needs?

| Not at all |  |  |  | Extremely |
| --- | --- | --- | --- | --- |
| 0 | 1 | 2 | 3 | 4 |

1. How readily can you put the sexual needs of your partner out of your thoughts?

| Not at all |  |  |  | Extremely |
| --- | --- | --- | --- | --- |
| 0 | 1 | 2 | 3 | 4 |

1. How high a priority for you is meeting the sexual needs of your partner?

| Not at all |  |  |  | Extremely |
| --- | --- | --- | --- | --- |
| 0 | 1 | 2 | 3 | 4 |

1. How easily could you accept not meeting your partner's sexual needs?

| Not at all |  |  |  | Extremely |
| --- | --- | --- | --- | --- |
| 0 | 1 | 2 | 3 | 4 |

1. How likely are you to sacrifice your own needs to meet the sexual needs of your partner?

| Not at all |  |  |  | Extremely |
| --- | --- | --- | --- | --- |
| 0 | 1 | 2 | 3 | 4 |

1. How happy do you feel when satisfying your partner's sexual needs?

| Not at all |  |  | |  | Extremely |
| --- | --- | --- | --- | --- | --- |
| 0 | 1 | 2 | 3 | | 4 |

**Measure of Unmitigated Sexual Communion.**

The measure of unmitigated sexual communion (USC) was adapted more recently, but in a sample of 101 community couples (sample used in Study 3 of Day, Muise, Joel, & Impett, 2015), we assessed USC in addition to SCS. In this sample, the measure of USC was reliable with a Cronbach’s alpha of .80.

USC is also correlated with general unmitigated communion at r = .33 (*p* < .001) and with SCS at r = .51 (*p* < .001) (in data mentioned above) and in a published study that assessed SCS and USC at the daily level at .35 (*p* < .001; Impett, Muise, & Harasymchuk, 2018), demonstrating convergent validity. As evidence of construct validity, whereas on days when people engage in sex and are high in SCS, they report higher sexual desire, when people engage in sex and are higher in USC, they do not report higher desire for their partner (Impett et al., 2018), suggesting that they are not meeting their partner’s sexual needs out of a genuine interest and motivation. As additional evidence of construct validity, in a clinical sample of couples coping with a sexual dysfunction, on days when people engage in sex and are higher in USC, they report higher personal sexual distress (Muise, Bergeron, Impett, & Rosen, 2018), which suggests that their communal motivation to meet their partner’s needs is not in line with genuine values and interests and instead may be self-neglecting and distressing.

**Unmitigated Sexual Communion (7 items).**

**Instructions**: Using the scale below, choose the number that indicates the extent to which you agree or disagree. Think of your relationship with your romantic partner when responding to each statement (1= strongly disagree to 5=strongly agree)

1. I always place my partner’s sexual needs above my own.

2. For me to be happy with my sex life, I need my partner to be happy.

3. I won’t be able to sleep if I think my partner is not sexually satisfied.

4. It is impossible for me to satisfy my own sexual needs if they interfere with the needs of my partner.

5. I can’t say no when my partner asks me to meet a sexual need of theirs.

6. Even when exhausted, I make sure I meet my partner’s sexual needs.

7. I often worry about my partner being unsatisfied with our sex life.

**Sexual Goals—Approach and Avoidance (partner-focused) (12 items).**

Please rate the importance of the following factors in influencing why you typically engage in sex with your partner from 1 = *not at all important* to 7 = *extremely important*).

# To please my partner.

# To promote intimacy in my relationship.

# To express love for my partner.

# To feel emotionally closer to my partner.

# To experience pleasure with my partner.

# To add excitement to my relationship.

# To prevent my partner from falling out of love with me.

# To prevent my partner from losing interest in me.

# To avoid having to decline a partner’s request.

# To prevent my partner from becoming upset.

# To avoid conflict in my relationship.

# To prevent my partner from getting angry at me.

**Sexual Desire—Sexual Desire Inventory-2 (SDI-2) (14 items).**

This questionnaire asks about your level of sexual desire. By desire, we mean INTEREST IN or WISH FOR SEXUAL ACTIVITY. For each item, please circle the number that best shows your thoughts and feelings.

- 1. During the last month, how often would you have liked to engage in sexual activity with your partner (for example, touching each other’s genitals, giving or receiving oral stimulation, intercourse, etc.)?
     1. Not at all
     2. Once a month
     3. Once every two weeks
     4. Once a week
     5. Twice a week
     6. 3 to 4 times a week
     7. Once a day
     8. More than once a day
  2. During the last month, how often have you had sexual thoughts involving your partner?
     1. Not at all
     2. Once a month
     3. Once every two weeks
     4. Once a week
     5. Twice a week
     6. 3 to 4 times a week
     7. Once a day
     8. More than once a day
  3. When you have sexual thoughts, how strong is your desire to engage in sexual behaviour with a partner?

| 0 | 1 | 2 | 3 | 4 | 5 | 6 | 7 | 8 |
| --- | --- | --- | --- | --- | --- | --- | --- | --- |
| No Desire |  |  |  |  |  |  |  | Strong Desire |

- 1. When you first see an attractive person, how strong is your sexual desire?

| 0 | 1 | 2 | 3 | 4 | 5 | 6 | 7 | 8 |
| --- | --- | --- | --- | --- | --- | --- | --- | --- |
| No Desire |  |  |  |  |  |  |  | Strong Desire |

- 1. When you spend time with an attractive person (for example, at work or school), how strong is your sexual desire?

| 0 | 1 | 2 | 3 | 4 | 5 | 6 | 7 | 8 |
| --- | --- | --- | --- | --- | --- | --- | --- | --- |
| No Desire |  |  |  |  |  |  |  | Strong Desire |

- 1. When you are in romantic situations (such as a candle lit dinner, a walk on the beach, etc.), how strong is your sexual desire?

| 0 | 1 | 2 | 3 | 4 | 5 | 6 | 7 | 8 |
| --- | --- | --- | --- | --- | --- | --- | --- | --- |
| No Desire |  |  |  |  |  |  |  | Strong Desire |

- 1. How strong is your desire to engage in sexual activity with a partner?

| 0 | 1 | 2 | 3 | 4 | 5 | 6 | 7 | 8 |
| --- | --- | --- | --- | --- | --- | --- | --- | --- |
| No Desire |  |  |  |  |  |  |  | Strong Desire |

- 1. How important is it for you to fulfil your sexual desire through activity with a partner?

| 0 | 1 | 2 | 3 | 4 | 5 | 6 | 7 | 8 |
| --- | --- | --- | --- | --- | --- | --- | --- | --- |
| Not At All Important |  |  |  |  |  |  |  | Extremely Important |

- 1. Compared to other people of your age and sex, how would you rate your desire to behave sexually with a partner?

| 0 | 1 | 2 | 3 | 4 | 5 | 6 | 7 | 8 |
| --- | --- | --- | --- | --- | --- | --- | --- | --- |
| Much Less Desire |  |  |  |  |  |  |  | Much More Desire |

- 1. During the last month, how often would you have liked to behave sexually by yourself (for example, masturbating, touching your genitals, etc.)?
     1. Not at all
     2. Once a month
     3. Once every two weeks
     4. Once a week
     5. Twice a week
     6. 3 to 4 times a week
     7. Once a day
     8. More than once a day
  2. How strong is your desire to engage in sexual behaviour by yourself?

| 0 | 1 | 2 | 3 | 4 | 5 | 6 | 7 | 8 |
| --- | --- | --- | --- | --- | --- | --- | --- | --- |
| No Desire |  |  |  |  |  |  |  | Strong Desire |

- 1. How important is it for you to fulfil your desires to behave sexually by yourself?

| 0 | 1 | 2 | 3 | 4 | 5 | 6 | 7 | 8 |
| --- | --- | --- | --- | --- | --- | --- | --- | --- |
| Not At All Important |  |  |  |  |  |  |  | Extremely Important |

- 1. Compared to other people if your age and sex, how would you rate your desire to behave sexually by yourself?

| 0 | 1 | 2 | 3 | 4 | 5 | 6 | 7 | 8 |
| --- | --- | --- | --- | --- | --- | --- | --- | --- |
| Much Less Desire |  |  |  |  |  |  |  | Much More Desire |

- 1. How long could you go comfortably without having sexual activity of some kind?
     1. Forever
     2. A year to two
     3. Several months
     4. A month
     5. A few weeks
     6. A week
     7. A few days
     8. One day
     9. Less than one day

#

# S6 Fig. Global Measure of Sexual Satisfaction (5 items).

**Instructions:** How would you describe your **overall sexual relationship** with your partner? For each pair of words, select the number that best describes your sexual relationship.

| Very bad |  |  |  |  |  | Very good |
| --- | --- | --- | --- | --- | --- | --- |
| 1 | 2 | 3 | 4 | 5 | 6 | 7 |

| Very unpleasant | |  |  |  | Very pleasant | |
| --- | --- | --- | --- | --- | --- | --- |
| 1 | 2 | 3 | 4 | 5 | 6 | 7 |

| Very negative | |  |  |  | Very positive | |
| --- | --- | --- | --- | --- | --- | --- |
| 1 | 2 | 3 | 4 | 5 | 6 | 7 |

| Very unsatisfying | |  |  |  | Very satisfying | |
| --- | --- | --- | --- | --- | --- | --- |
| 1 | 2 | 3 | 4 | 5 | 6 | 7 |

| Very worthless | |  |  |  | Very valuable | |
| --- | --- | --- | --- | --- | --- | --- |
| 1 | 2 | 3 | 4 | 5 | 6 | 7 |

# Sexual Distress—Female Sexual Distress Scale-Revised (FSDS-R) (13 items).

**Instructions**: Below is a list of feelings and problems that women sometimes have concerning their sexuality. Please read each item carefully, and circle the number that best describes HOW OFTEN THAT PROBLEM HAS BOTHERED YOU OR CAUSED YOU DISTRESS DURING THE **PAST 30 DAYS** INCLUDING TODAY.

**How often did you feel:**

|  |  | Never | Rarely | Occasionally | Frequently | Always |
| --- | --- | --- | --- | --- | --- | --- |
| 1 | Distressed about your sex life | 1 | 2 | 3 | 4 | 5 |
| 2 | Unhappy about your sexual relationship | 1 | 2 | 3 | 4 | 5 |
| 3 | Guilty about sexual difficulties | 1 | 2 | 3 | 4 | 5 |
| 4 | Frustrated by your sexual problems | 1 | 2 | 3 | 4 | 5 |
| 5 | Stressed about sex | 1 | 2 | 3 | 4 | 5 |
| 6 | Inferior because of sexual problems | 1 | 2 | 3 | 4 | 5 |
| 7 | Worried about sex | 1 | 2 | 3 | 4 | 5 |
| 8 | Sexually inadequate | 1 | 2 | 3 | 4 | 5 |
| 9 | Regrets about your sexuality | 1 | 2 | 3 | 4 | 5 |
| 10 | Embarrassed about sexual problems | 1 | 2 | 3 | 4 | 5 |
| 11 | Dissatisfied with your sex life | 1 | 2 | 3 | 4 | 5 |
| 12 | Angry about your sex life | 1 | 2 | 3 | 4 | 5 |
| 13 | Bothered by low sexual desire | 1 | 2 | 3 | 4 | 5 |

# Sexual Frequency (9 items).

1. During the past 4 weeks, how often did you and your partner engage in the following behaviours:

| 0 = | Not at all | 4 = | 4-5 times a week |
| --- | --- | --- | --- |
| 1 = | Once or twice | 5 = | Once a day |
| 2 = | Once a week | 6 = | More than once a day |
| 3 = | 2-3 times a week |  |  |

| Kissing | 0 | 1 | 2 | 3 | 4 | 5 | 6 |
| --- | --- | --- | --- | --- | --- | --- | --- |
| Caressing/Touching | 0 | 1 | 2 | 3 | 4 | 5 | 6 |
| Oral sex (giving to partner) | 0 | 1 | 2 | 3 | 4 | 5 | 6 |
| Oral sex (receiving from partner) | 0 | 1 | 2 | 3 | 4 | 5 | 6 |
| Giving manual stimulation (touching or massaging your partner’s genitals) | 0 | 1 | 2 | 3 | 4 | 5 | 6 |
| Receiving manual stimulation (your partner touching or massaging your genitals) | 0 | 1 | 2 | 3 | 4 | 5 | 6 |
| Manual stimulation (masturbation) (alone) | 0 | 1 | 2 | 3 | 4 | 5 | 6 |
| Sexual intercourse with vaginal penetration | 0 | 1 | 2 | 3 | 4 | 5 | 6 |
| Sexual intercourse with anal penetration | 0 | 1 | 2 | 3 | 4 | 5 | 6 |
